# The impact of stroke on the ability to live an independent life at old age: a community-based cohort study of Swedish men

**DOI:** 10.1186/s12877-023-03817-1

**Published:** 2023-03-06

**Authors:** Elias Lindvall, Kristin Franzon, Erik Lundström, Lena Kilander

**Affiliations:** 1 Department of Medical Sciences, Neurology, Uppsala University, Uppsala Academic Hospital, Neurological Reception, Sjukhusvägen, 75185 Uppsala, Sweden; 2 Department of Public Health and Caring Sciences, Geriatrics, Uppsala University, Uppsala, Sweden

**Keywords:** Stroke, Dementia, Community, Cohort, Independency, Walking, Activities of daily living

## Abstract

**Introduction:**

Few studies with controls from the same cohort have investigated the impact of stroke on the ability to live an independent life at old age. We aimed to analyze how great an impact being a stroke survivor would have on cognition and disability. We also analyzed the predictive value of baseline cardiovascular risk factors.

**Methods:**

We included 1147 men, free from stroke, dementia, and disability, from the Uppsala Longitudinal Study of Adult Men, between 69–74 years of age. Follow-up data were collected between the ages of 85–89 years and were available for 481 of all 509 survivors. Data on stroke diagnosis were obtained through national registries. Dementia was diagnosed through a systematic review of medical charts and in accordance with the current diagnostic criteria. The primary outcome, preserved functions, was a composite outcome comprising four criteria: no dementia, independent in personal activities of daily living, ability to walk outside unassisted, and not living in an institution.

**Results:**

Among 481 survivors with outcome data, 64 (13%) suffered a stroke during the follow-up. Only 31% of stroke cases, compared to 72% of non-stroke cases (adjusted OR 0.20 [95% CI 0.11–0.37]), had preserved functions. The chance of being free of dementia was 60% lower in the stroke group, OR 0.40 [95% CI 0.22–0.72]. No cardiovascular risk factors were independently able to predict preserved functions among stroke cases.

**Conclusion:**

Stroke has long lasting consequences for many aspects of disability at very high age.

**Supplementary Information:**

The online version contains supplementary material available at 10.1186/s12877-023-03817-1.

## Introduction

Stroke is the third leading cause of disability worldwide [1]. Due to a higher proportion of patients being treated in stroke units and more effective secondary prevention, more people survive stroke, even in very high age, leaving an increasing number of individuals with varying degrees of sequelae [2]. Few studies have specifically addressed the impact of stroke on disability in people over 80 years. Although predictors of disability affecting daily activities after stroke have been studied, follow-up over several years seems to be scarce [3].

Approximately, 10–30% of stroke victims develop dementia within one year [4]. Stroke is a strong contributor to vascular dementia [5] and may accelerate neurodegeneration in Alzheimer’s disease [6].

Other common consequences of stroke in the oldest old are loss of the ability to walk outdoors and the need for assisted care facilities. These aspects have a negative impact on independency and quality of life [7], as well as societal costs. However, the extent to which stroke per se contributes to disability is not fully clear, since only a few longitudinal studies have compared subjects with and without stroke from the same population-based cohort.

The Uppsala Longitudinal Study of Adult Men (ULSAM) is a community-based cohort of Swedish men, who have been followed for more than four decades with a focus on age-associated disorders and independent aging [8, 9].

The aim of this study was to explore how stroke after 70 years of age affects the ability to live an independent life, in men surviving above age 85. Further, we analyzed whether the cardiovascular risk profile at age 70 predicted the long-term functional independence following the stroke. Our primary outcome – preserved functions – was defined as independency in personal activities of daily living (PADL), absence of dementia, being able to walk outdoors on your own, and living in your own home.

## Methods

### Study design and population

**Fig. 1:**
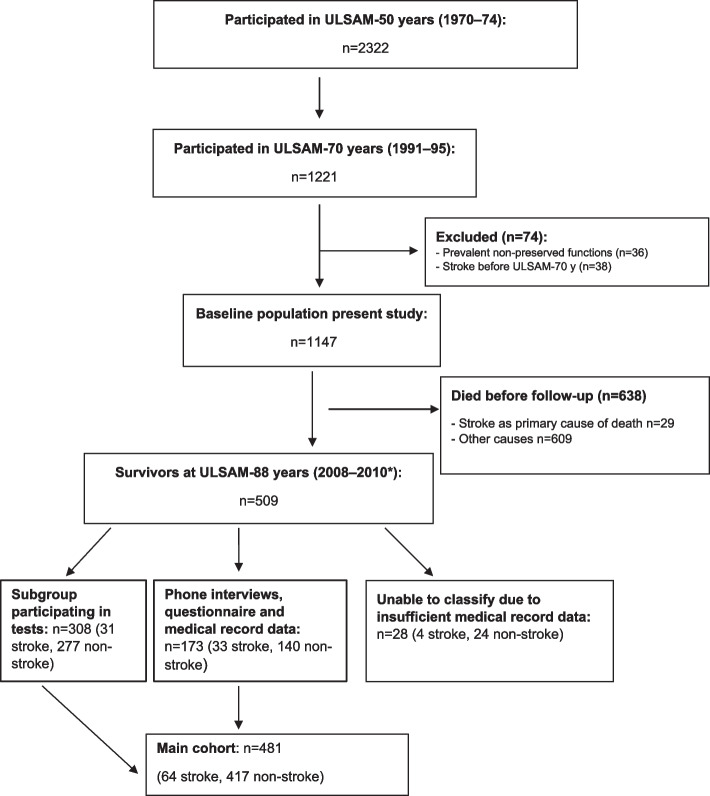
Flow chart of the study population.*ULSAM-88 years was finished in December 2009. Data from phone interviews, questionnaires and medical records were gathered up until March 2010

All men living in the county of Uppsala, born between 1920–1924 (*n* = 2841), were offered to participate in the first ULSAM investigation during 1970–1974; specifically, 82% (2322/2841) accepted the invitation. For the present study, 1221 men (mean age 71 years) in the third ULSAM investigation cycle (ULSAM-70) were eligible for inclusion (Fig. 1). We excluded individuals with a prior stroke diagnosis in the national in-patient registry (*n* = 38) and those who did not meet the criteria for preserved functions (*n* = 36), through a systemic review of questionnaires and medical charts. Hence, a total of 1147 men (94%) were included in our baseline cohort.

At follow-up after approximately sixteen years, a total of 638 individuals (56%) had died; moreover, 28 survivors, representing 6% of all stroke cases and 5% of non-stroke cases, could not be classified regarding functions due to insufficient data in their medical charts. These men had only a few visits to general practitioners and hospitals. Furthermore, 481 individuals (42%) had sufficient primary outcome data (‘main cohort’) to be classified regarding preserved functions or not. Out of these, 308 men (64%) also participated in functional tests, such as gait speed, cognitive tests, and blood sampling (‘subgroup participating in tests’).

The outcome data were collected at the sixth ULSAM investigation face-to-face (ULSAM-88) and through telephone interviews and reviews of medical charts.

### Baseline data

The ULSAM data collection procedure is described in detail at the website (https://www.pubcare.uu.se/ulsam/Database). Baseline data were collected between August 1991 and May 1995 (for details, see additional file 1). Data on educational level, marital status, physical activity, smoking habits, living conditions, and PADL functions were collected through interviews and questionnaires. Research nurses recorded the blood pressure, electro-cardiogram, drew blood samples, and calculated the body mass index (BMI). Fasting blood glucose values were obtained from an oral glucose intolerance test. Diabetes [10] and hypertension [11] were defined in accordance with the international criteria. Drug prescription data were available through the Swedish prescribed drug registry. The Mini Mental State Examination (MMSE) [12] enhanced cued recall [13], temporal orientation [13] and Trail Making Test B (TMTB) [14] were administered. Charlson comorbidity index was calculated based on the comorbidities available in the International classification of diseases (ICD) from the Swedish in-patient registry [15, 16].

### Stroke

The first-ever stroke diagnoses were retrieved from the in-patient registry and defined as hospitalization with any stroke diagnosis (ICD version 8–10, see Additional file 1); before follow-up date in ULSAM-88 or before March 10, 2010 (participants with only medical chart data).

### Outcome data

All 509 surviving ULSAM participants were invited to ULSAM-88 at the Geriatric department, Uppsala University hospital between September 2008 and December 2009; and 308 participated at the hospital or had a home visit by a research nurse (‘subgroup participating in tests’). For individuals who did not participate in ULSAM-88 (201/509), phone interviews were conducted, as well as questionnaires and a review of medical charts from January 2010 to March 2010. The diagnostic workup for dementia was made according to standard clinical procedures at either the Memory Clinic (mandatory for a diagnosis of Lewy body dementia and frontotemporal dementia) or in primary care, and cases were identified by reviews of the medical records. Caregivers’ descriptions of cognitive problems, impact on IADL, MMSE and Clock test were always included. The cognitive tests administered to participants in ULSAM-88 were on separate occasions, and were not included in these diagnostic procedures. Dementia was diagnosed by two experienced geriatricians (LK and KF) who independently examined all records and cognitive data available at the end of ULSAM-88/March 2010. Established diagnostic criteria and neuroradiology were used to classify cases as Alzheimer’s disease [17], vascular dementia [18], mixed Alzheimer’s and vascular dementia, Lewy body dementia/Parkinson dementia [19, 20], or frontotemporal dementia [21]. A diagnosis of unspecified dementia was set in cases with insufficient information. A third geriatrician was consulted in case of discordance, and a majority decision was made*.*

A questionnaire was administered to acquire data on living conditions, everyday physical activity, and PADL-function. For test participants, research nurses collected data on gait speed [22], ability to stand without support for 30 seconds [23], and Timed Up-and-Go Test [24]. Further, they administered the MMSE [12], enhanced cued recall (16 items) and verbal fluency (animals) from the 7 Minute Screen [13], the Geriatric Depression Scale-20 [25]; and a 0–100% visual analogue quality of Life scale [26].

### Outcome definitions

The primary outcome – preserved functions – was defined as fulfilling the following 4 criteria, through data from questionnaires, medical charts, or phone interviews:

1. Absence of a dementia diagnosis
2. Able to walk outdoors unassisted
3. Independency in personal activities of daily living
4. Living in your own home, i.e., not living in an institution or care facility

Absence of a dementia diagnosis was included as a separate outcome for the predictive value of baseline variables analysis.

### Statistical analysis

Differences between the stroke and non-stroke participants were analyzed with Students t–test or chi-square, depending on the level of measurement for the predictor variable. When assumptions of chi-square were not met, Fisher’s exact test (nominal variables) or Likelihood ratio (ordinal variables) were used. Logistic regression was used to calculate the odds ratios (OR) and 95% confidence intervals (CI) for the outcomes. All outcomes were adjusted for age. Preserved functions and dementia were also adjusted for education. The outcome differences for the subgroup participating in the tests were analyzed using T-test or chi-square. For secondary outcomes, p-values were adjusted for age using ANCOVA-test. Additionally, cognitive test data were adjusted for education using ANCOVA.

Logistic regression was used to determine the predictive value of baseline variables for preserved functions and non-dementia. The analysis was performed separately for each individual baseline predictor, and the results were presented as OR with 95% CI. Continuous variables were analyzed as Z-scores so that the odds ratios would reflect increases in the standard deviation (SD).

A sensitivity analysis was performed to compare the rate of stroke in subjects with insufficient outcome data and the main cohort. All statistical analyses were performed using IBM SPSS Statistics Software (version 27 for PC; IBM Corp., NY, USA). Author ELi had access to all data, takes responsibility for data integrity and performed all statistical analyses.

### Ethical considerations

ULSAM collected data with ethical approval from the regional ethics board in Uppsala (1990–10-10, Dnr 251/90 and 2008–01-23, Dnr 2007/338). All participants had given their informed consent.

## Results

During follow-up, a total of 64 participants (13%) suffered a stroke. Among these, 8% had an intracerebral hemorrhage, and 2% had a subarachnoid hemorrhage. The mean time from the first-ever stroke to follow-up was 5.7 years (SD = 4.8 years), and one-third (21/64) had a stroke before age 80. Twenty-six participants (41%) had multiple hospital admissions due to the stroke. The proportion of stroke cases was slightly lower in the subgroup participating in tests (10%) than in the main cohort (13%). Baseline variables in the main cohort are presented in Table 1. As expected, the stroke cases had a higher systolic blood pressure and prevalence of hypertension, as well as atrial fibrillation. These differences were consistent in the subgroup participating in the tests, except for atrial fibrillation (data not shown).

**Table 1:** Baseline characteristics of subsequent stroke and non-stroke cases

		**Main cohort (*n* = 481)**		
**Variables:**		**Stroke (*n* = 64)**	**Non-stroke (*n* = 417)**	***p*-value**
**Mean age ***years (SD)*	*n *= 481	71.1 (0.6)	70.9 (0.6)	0.034
**Education**	*n* = 481			0.354
** < 8 years ***n*		39 (61%)	217 (52%)	
** 8–13 years ***n*		15 (23%)	131 (31%)	
** > 13 years ***n*		10 (16%)	69 (17%)	
**Living with a partner**	*n* = 466	55 (90%)	355 (88%)	0.574
**Smoking**	*n *= 467			0.942
** Current ***n*		8 (13%)	58 (15%)	
** Former ***n*		31 (50%)	200 (49%)	
** Never ***n*		23 (37%)	147 (36%)	
**Leisure time physical activity**	*n* = 458			0.863
** Low ***n*		19 (32%)	133(33%)	
** High ***n*		40 (68%)	266 (67%)	
**Systolic BP mm Hg ***(SD)*	*n* = 480	150 (19)	144 (18)	0.029
**Diastolic BP mm Hg ***(SD)*	*n* = 480	85 (10)	83 (9)	0.242
**HDL cholesterol mmol/L ***(SD)*	*n* = 478	1.31 (0.36)	1.31 (0.34)	0.856
**LDL cholesterol mmol/L ***(SD)*	*n* = 475	3.99 (0.91)	3.87 (0.88)	0.334
**BMI *kg/m***^***2***^ (SD)	*n* = 480	26.2 (3.4)	26.2(3.1)	0.982
**Diabetes ***n*	*n* = 480	12 (19%)	52 (13%)	0.152
**Hypertension ***n*	*n* = 480	48 (76%)	253 (61%)	0.018
**Atrial fibrillation*** n*	*n* = 454	5 (9%)	9 (2%)	0.023
**MMSE score ***(SD)*	*n* = 389	29 (1.3)	29 (1.3)	0.870
**TMT B seconds ***(SD)*	*n* = 423	116 (43)	111(44)	0.397
**Charlson comorbidity index**	*n* = 481			
** Index value 0*** n*		49 (77%)	338 (81%)	
** Index value 1 ***n*		13 (20%)	69 (17%)	
** Index value 2–4 ***n*		2 (3%)	10 (2%)	

Values are absolute numbers or means. Percentages represent proportions of all individuals with available data. *SD* Standard deviation. *BP* Blood pressure. *MMSE* Mini Mental State Examination. *TMT B* Trail making test B. *BMI* Body mass index. *HDL* High-density lipoprotein. *LDL* Low-density lipoprotein. Students t-test and chi-square were used for *p*-values

Only one-third of the stroke victims (31%), and more than two-thirds (72%) of the non-stroke participants fulfilled the criteria for preserved functions (Table 2). Stroke decreased the odds of having preserved functions at age 85–89 by 80%, OR 0.20 (95% CI 0.11–0.37). The proportion of individuals without dementia was 84% in men without a stroke and 63% among those afflicted by a stroke, OR 0.40 (95% CI 0.22–0.72). For stroke cases with dementia, the frequency of either vascular dementia or mixed Alzheimer’s and vascular dementia was 71%. Out of the four components, the ability to walk outdoors without assistance was most often affected in stroke. The subgroup participating in tests had higher rates of preserved functions in both non-stroke and stroke individuals, reflecting the higher participation rate among unimpaired subjects. There was no difference in Enhanced cued recall or temporal orientation. Stroke individuals had lower scores in the quality of life scale (60 vs. 72), verbal fluency test (13 points vs. 16 points), and higher scores in the Geriatric Depression Scale-20 (3.9 points vs. 2.7 points).

**Table 2:** Outcome measurements in incident stroke and non-stroke participants

**Primary outcome**					
	**Main cohort (*n* = 481)**				
**Variables:**	**Total**	**Stroke**	**Non-stroke**	**Unadjusted OR (95%CI)**	**Adjusted OR (95%CI)**
		***n* = 64,13%**	***n* = 417, 87%**		
**Mean age at first ever stroke ***years (SD)*		81.7 (4.8)			
**Preserved Functions ***n/total n*	322/481 (67%)	20/64 (31%)	302/417 (72%)	0.17 (0.01–0.31)	0.20 (0.11–0.37)^a^
Non dementia *n/total n*	388/481 (81%)	40/64 (63%)	348/417 (84%)	0.33 (0.19–0.58)	0.40 (0.22–0.72)^a^
PADL independent *n/total n*	345/423 (72%)	24/47 (51%)	321/376 (85%)	0.18 (0.09–0.34)	0.19 (0.10–0.37)^b^
Walking unassisted outdoors *n/total n*	353/430 (73%)	22/47 (47%)	331/383 (86%)	0.14 (0.07–0.26)	0.16 (0.08–0.31)^b^
Living at home *n/total n*	400/470 (83%)	37/60 (62%)	363/410 (89%)	0.21 (0.11–0.38)	0.24 (0.13–0.45)^b^
	**Subgroup participating in tests (*n* = 308)**				
**Variables:**		**Stroke**	**Non-stroke**	**Unadjusted OR (95%CI)**	**Adjusted OR (95%CI)**
**Preserved Functions ***n/total n*		17/31 (55%)	235/277 (85%)	0.22 (0.10–0.47)	0.25 (0.11–0.56)^a^
Non dementia *n/total n*		26/31 (84%)	259/277 (94%)	0.36 (0.12–1.05)	0.37 (0.13–1.12)^a^
PADL independent *n/total n*		21/31 (68%)	252/277 (91%)	0.21 (0.09–0.49)	0.24 (0.10–0.58)^b^
Walking unassisted outdoors *n/total n*		19/31 (61%)	258/277 (93%)	0.12 (0.05–0.28)	0.13 (0.06–0.32)^b^
Living at home *n/total n*		25/31 (81%)	270/277 (98%)	0.11 (0.03–0.35)	0.12 (0.04–0.38)^b^
**Secondary outcomes**					
	**Subgroup participating in tests (*n* = 308)**				
**Variables:**		**Stroke**	**Non-stroke**	**Unadjusted** ***p*-value**	**Adjusted** ***p*-value**
		***n* = 31, 10%**	***n *= 277, 90%**		
**Gait speed ***m/s (SD)*	*n* = 251	1.26(0.38)	1.37(0.32)	0.337	0.348^b^
**Able to stand without support ***n*	*n *= 286	23 (100%)	262 (100%)	1.000	0.998^b^
**Timed up and go** *seconds (SD)*	*n* = 250	17 (12.2)	14 (7.1)	0.381	0.210^b^
**MMSE ***score (SD)*	*n* = 306	25.8 (5.0)	27.0 (3.2)	0.215	0.205^a^
**Verbal fluency ***score (SD)*	*n* = 303	13 (5.6)	16 (5.3)	0.035	0.046^a^
**Geriatric depression scale-20 ***score (SD)*	*n* = 300	3.9 (2.6)	2.7 (2.5)	0.031	0.036^b^
**Quality of life scale ***score (SD)*	*n* = 288	60 (20)	72 (16)	0.007	0.002^b^

Values are absolute numbers or means. Percentages represent proportions of all individuals with available data. Logistic regression was used to derive Odds Ratios (OR). *PADL* Personal activities of daily living. *MMSE* Mini mental state examination. Students t-test and chi-square were used. ANCOVA was used for adjusted *p*-values

^a^Adjusted for age and education

^b^Adjusted for age

No baseline variables were independent predictors of the preserved functions (Table 3) or dementia (Table 4) in the stroke group. However, among non-stroke individuals, hypertension (OR 0.53 [95% CI 0.33–0.84]) and slower performance on TMT B (OR 0.58 [95% CI 0.45–0.75]) were associated with lower odds, whereas higher MMSE scores (OR 1.47 [95% CI 1.11–1.93]) were associated with a greater chance of having preserved functions. Furthermore, hypertension (0.45 [95% CI 0.25–0.80]) and slow performance on the TMT B (OR 0.58 [95% CI 0.44–0.77]) were associated with lower odds of non-dementia. High MMSE scores (OR 1.78 [95% CI 1.31–2.44]) predicted higher odds of non-dementia.

**Table 3:** Baseline variables predicting Preserved Functions

	**Stroke cases (*n* = 64)**				**Non-stroke cases (*n* = 417)**			
Variables:		**PF (*n* = 20)**	**Non-PF (*n* = 44)**	**Unadjusted OR (95% CI)**		**PF (*n* = 302)**	**Non-PF (*n* = 115)**	**Unadjusted OR (95% CI)**
	Available data				Available data			
**Education**	*n* = 64				*n* = 417			
** < 8 years ***n*		12 (60%)	27 (61%)	ref		157 (52%)	60(52%)	Ref
** 8–13 years ***n*		5 (25%)	10 (23%)	1.13 (0.32–4.01)		93 (31%)	38 (33%)	0.94 (0.58–1.51)
** > 13 years ***n*		3 (15%)	7 (16%)	0.96 (0.21–4.38)		52 (17%)	17 (15%)	1.17 (0.63–2.18)
**Living with a partner**	*n* = 61	19 (95%)	36 (88%)	2.64 (0.29–24.24)	*n* = 405	258 (87%)	97 (90%)	0.75 (0.37–1.52)
**Smoking**	*n* = 62				*n* = 405			
** Current ***n*		2 (10%)	6 (14%)	ref		40 (14%)	18 (16%)	Ref
** Former ***n*		10 (50%)	21 (50%)	1.43 (0.24–8.38)		147 (50%)	53 (48%)	1.25 (0.66–2.36)
** Never ***n*		8 (40%)	15 (36%)	1.60 (0.26–9.83)		107 (36%)	40 (36%)	1.20 (0.62–2.34)
**Leisure time physical activity**	*n* = 59				*n* = 399			
** Low ***n*		4 (21%)	15(38%)	ref		99 (34%)	34 (32%)	Ref
** High ***n*		15 (79%)	25 (63%)	2.25 (0.63–8.05)		194 (66%)	72 (68%)	0.93 (0.58–1.49)
**Systolic BP mm Hg ***(SD)*	*n* = 63	148 (17)	151 (21)	0.87 (0.52–1.46)	*n* = 417	143 (18)	147 (17)	0.77 (0.62–0.97)
**Diastolic BP mm Hg ***(SD)*	*n* = 63	83 (9)	85 (10)	0.75 (0.44–1.28)	*n* = 417	83 (9)	84 (9)	0.82 (0.66–1.02)
**HDL cholesterol mmol/L ***(SD)*	*n *= 63	1.39 (0.36)	1.27 (0.34)	1.37 (0.81–2.32)	*n* = 414	1.29 (0.33)	1.31 (0.34)	0.94 (0.76–1.18)
**LDL cholesterol mmol/L ***(SD)*	*n* = 63	4.09 (0.97)	3.95 (0.89)	1.17 (0.69–1.98)	*n* = 412	3.88 (0.90)	3.87 (0.84)	1.01 (0.81–1.26)
**BMI *kg/m***^***2***^ (SD)	*n *= 63	25.6 (3.4)	26.4(3.5)	0.76 (0.43–1.34)	*n* = 417	26.1 (2.9)	26.6 (3.6)	0.83 (0.65–1.04)
**Diabetes ***n*	*n* = 63	3 (15%)	9 (21%)	0.67 (0.16–2.79)	*n* = 417	34 (11%)	18(16%)	0.68 (0.37–1.27)
**Hypertension ***n*	*n* = 63	15 (76%)	33 (77%)	0.91 (0.26–3.13)	*n* = 417	171 (57%)	82 (71%)	0.53 (0.33–0.84)
**Atrial fibrillation*** n*	*n* = 58	1 (5%)	4 (10%)	0.49 (0.05–4.68)	*n* = 396	7 (2%)	2 (2%)	1.30 (0.27–6.37)
**MMSE score ***(SD)*	n = 52	29 (1.0)	28 (1.4)	1.80 (0.80–4.05)	n = 337	29 (1.1)	28 (1.6)	1.47 (1.11–1.93)
**TMT B seconds ***(SD)*	n = 60	115 (42)	117(44)	0.95 (0.51–1.77)	n = 363	105 (40)	127 (49)	0.58 (0.45–0.75)
**Charlson comorbidity index**	n = 64				n = 417			
** Index value 0*** n*		16 (80%)	33 (75%)	0.49 (0.03–8.26)		244 (81%)	94 (82%)	1.73 (0.48–6.27)
** Index value 1 ***n*		3 (15%)	10 (23%)	0.30 (0.01–6.38)		52 (17%)	17 (15%)	2.04 (0.51–8.09)
** Index value 2–4 ***n*		1 (5%)	1 (2%)	ref		6 (2%)	4 (4%)	Ref

Odds ratio (OR) reflect increases in standard deviation (SD) of the variable. Available data was used to calculate percentages. Logistic regression was used. *PF *Preserved functions, *BP* Blood pressure. *MMSE* Mini mental state examination. *TMT B* Trail Making Test B. *BMI* Body mass index. *HDL*  High-density lipoprotein. *LDL* Low-density lipoprotein

**Table 4:** Baseline variables predicting non-dementia

	**Stroke cases (*n* = 64)**				**Non-stroke cases (*n* = 417)**			
**Variables:**		**Non-dementia (*n* = 40)**	**Dementia (*n* = 24)**	**Unadjusted OR (95% CI)**		**Non-dementia (*n* = 348)**	**Dementia (*n* = 69)**	**Unadjusted OR (95% CI)**
	**Available data**				**Available data**			
**Education**	*n* = 64				*n* = 417			
** < 8 years ***n*		25 (63%)	14 (58%)	ref		181 (52%)	36 (52%)	ref
** 8–13 years ***n*		8 (20%)	7 (29%)	0.64 (0.19–2.14)		109 (31%)	22 (32%)	0.99 (0.55–1.76)
** > 13 years ***n*		7 (18%)	3 (13%)	1.31 (0.29–5.87)		58 (17%)	11 (16%)	1.05 (0.50–2.19)
**Living with a partner**	*n* = 61	34 (90%)	15 (71%)	3.40 (0.84–13.84)	*n* = 405	297 (87%)	58 (91%)	0.70 (0.28–1.71)
**Smoking**	*n* = 62				*n* = 405			
** Current ***n*		4 (10%)	4 (17%)	ref		48 (14%)	10 (15%)	ref
** Former ***n*		21 (54%)	10 (44%)	1.10 (0.43–10.17)		166 (49%)	34 (50%)	1.02 (0.47–2.21)
** Never ***n*		14 (36%)	9 (39%)	1.56 (0.31–7.85)		123 (37%)	24 (35%)	1.07 (0.48- 2.40)
**Leisure time physical activity**	*n* = 59				*n* = 399			
** Low ***n*		10 (28%)	9(39%)	ref		117 (35%)	16 (25%)	Ref
** High ***n*		26 (72%)	14 (61%)	1.67 (0.55–5.07)		219 (65%)	47 (75%)	0.64 (0.35–1.17)
**Systolic BP mm Hg ***(SD)*	*n* = 63	152 (20)	147 (20)	1.27 (0.77–2.09)	*n* = 417	143 (18)	148 (17)	0.78 (0.60–1.01)
**Diastolic B Diastolic BP mm Hg** *(SD)*	*n* = 63	85 (9)	84 (11)	1.16 (0.70–1.91)	*n* = 417	83 (9)	84 (10)	0.88 (0.68–1.15)
**HDL cholesterol mmol/L ***(SD)*	*n* = 63	1.33 (0.37)	1.28 (0.33)	1.17 (0.70–1.94)	*n* = 415	1.30 (0.33)	1.33 (0.38)	0.91 (0.70–1.19)
**LDL cholesterol mmol/L ***(SD)*	*n* = 63	4.14 (0.94)	3.76 (0.82)	1.58 (0.90–2.77)	*n* = 412	3.88 (0.88)	3.85 (0.91)	1.04 (0.80–1.35)
**BMI *kg/m***^***2***^ (SD)	*n *= 61	26.3 (3.5)	26.0 (3.4)	1.11 (0.66–1.86)	*n* = 407	26.2 (3.1)	26.5 (3.4)	0.90 (0.68–1.19)
**Diabetes ***n*	*n* = 63	7 (20%)	5 (21%)	0.83 (0.23–2.99)	*n* = 417	43 (12%)	9(13%)	0.94 (0.44–2.03)
**Hypertension ***n*	*n* = 63	32 (82%)	16 (67%)	2.29 (0.70–7.43)	*n* = 417	201 (58%)	52 (75%)	0.45 (0.25–0.80)
**Atrial fibrillation*** n*	*n* = 58	4 (11%)	1 (5%)	2.42 (0.25–23.25)	*n* = 396	7 (2%)	2 (3%)	0.66 (0.13–3.23)
**MMSE score ***(SD)*	*n* = 52	29 (1.2)	28 (1.3)	1.43 (0.72–2.86)	*n* = 337	29 (1.2)	28 (1.9)	1.78 (1.31–2.44)
**TMT B seconds ***(SD)*	*n* = 60	115 (42)	117(44)	0.95 (0.51–1.77)	*n* = 363	105 (40)	127 (49)	0.58 (0.45–0.75)
**Charlson comorbidity index**	*n* = 64				*n *= 417			
** Index value 0*** n*		30 (75%)	19 (79%)	-		281 (81%)	57 (83%)	0.55 (0.07–4.41)
** Index value 1 ***n*		8 (20%)	5 (21%)	-		58 (17%)	11 (16%)	0.59 (0.07–5.10)
** Index value 2–4 ***n*		2 (5%)	0 (0%)	ref		9 (3%)	1 (1%)	ref

Odds ratio (OR) reflect increases in standard deviation (SD) of the variable. Available data was used to calculate percentages. Logistic regression was used. *PF *Preserved functions, *BP* Blood pressure. *MMSE* Mini mental state examination. *TMT B* Trail Making Test B. *BMI* Body mass index. *HDL* High-density lipoprotein. *LDL* Low-density lipoprotein

### Sensitivity analysis

The stroke rate was similar in individuals who were unclassifiable due to insufficient medical chart data, as in the main cohort (14% vs. 13%, *p* = 0.779).

## Discussion

In this community-based cohort of men aged 85–59 years, free from stroke and disability at baseline, stroke during the follow up period was associated with 80% lower odds of preserved functions, compared to stroke-free survivors. Stroke survivors had higher odds ratios for all four aspects of disability, i.e., dementia, dependency in PADL, loss of the ability to walk outdoors on your own, and institutionalization. These are vital for a person’s ability to pursue meaningful and joyous activities in their everyday life [7, 27].

Unsurprisingly, we found no other stroke study with a similar composite outcome; therefore, we will discuss cognition, loss of independence, and quality of life separately.

Dementia after stroke is by far the most studied. However, most reports are from hospital-based cohorts, with follow-up for 1–3 years. Moreover, they often lack a relevant control group, which is essential since other causes of functional impairment are common in very high age [28, 29].

To the best of our knowledge, only five previous studies have investigated the risk of dementia in stroke compared to stroke-free subjects from the same cohort [30–34]. In the Framingham study, with a mean age at entry of 79 years, stroke was associated with a twofold increase in the hazard ratio of dementia over 10 years of follow-up [30]. Cognitive impairment or dementia was present in 64% of the stroke survivors compared to 21% in the stroke-free subjects after 5 years of follow-up in the Canadian Study of Health and Aging [31]. The Rotterdam Scan Study included subjects with a mean age of 69 years, and the hazard ratio for dementia was doubled in those eleven percent who suffered a stroke during a mean of 7 years of follow-up [34]. In the Kungsholmen Study, all participants were more than 75 years at the time of inclusion and followed for 3 years. Both first-ever stroke (relative risk 2.4) and a history of stroke (relative risk 1.7) were associated with dementia [32]. Similar results are reported from the Icelandic MRI study [33]. In ULSAM, the OR for post-stroke dementia was 2.5. The overall prevalence of dementia was 19%, which is marginally lower than the expected prevalence in this age group [35].

Loss of independency in outdoor walking and managing PADL were more common than dementia in our study. Results from previous studies are inconclusive since the methods vary widely and follow-up periods are only for a few years. The modified Rankin Scale is probably the most common disability measurement in stroke trials [36]. Our outcome items are more precisely defined. Neurological deficits at onset, upper limb paresis, and high age are predictors of ADL function [3]. In a Swedish hospital-based cohort, one year after a first-ever stroke, one-fifth lived in nursing homes and more than one-third of the survivors were PADL dependent, i.e., less than in our cohort [37]. In Sweden, institutionalization is indicated when home care services are no longer able to provide the support needed, and it is not associated with socioeconomic status. The 5-year risk of institutionalization after stroke was 26% in the Oxford Vascular Study, which is lower than in our study (38%) [38]. This is probably explained by the fact that our participants were older at the time of follow-up (87 vs. 75 years).

We saw a negative impact on quality of life among those afflicted by stroke, as well as slightly higher ratings on the Geriatric Depression Scale. This shows that stroke certainly affects the well-being in those who survive beyond their eighties. Men with stroke had lower scores in verbal fluency but not in the MMSE. This may be because verbal fluency relies more on semantic memory, speech, executive function and cognitive speed than MMSE.

Although MMSE is not sensitive memory test, the difference in score might reflect a steeper cognitive decline for stroke survivors because of cerebrovascular disease [5, 39].

None of the baseline cardiovascular parameters were independent predictors of preserved functions in stroke cases. This is in line with previous studies where the addition of other cardiovascular parameters in a predictive model did not increase the general risk of dementia/cognitive decline post stroke [34, 40].

The limitations of this study include the lack of stroke specific data, such as stroke severity, infarct volume, and type of stroke. These are well known predictors for disability, at least for the first 5 years after a stroke [4, 41]. Generalizability may be affected due to recruitment from a single region and the lack of female participants; however, previous studies comparing the prevalence of post stroke cognitive impairment between men and women show no significant difference [34, 42]. We estimated approximately 100 stroke survivors, based on the rates of participation in ULSAM. However, we had fewer stroke cases, probably because many ULSAM participants have had their vascular risk factors addressed and treated since age 50. One major limitation is the small numbers participating in tests, although men in this age group are rare, and our study adds knowledge in this population. As a consequence the predictive value of baseline variables may have been underestimated.

The strengths include the fact that participants were included from a community-based population and free from stroke and disability at baseline. Data were collected prospectively, and register data allowed us to capture all hospitalized stroke cases. We were able to track the primary outcome in 94% of the survivors through phone interviews and medical chart reviews. The study design allowed us to compare OR between stroke and non-stroke subjects, which is important in high age where other causes of disability are frequent. Other major strengths of this study are the homogeneity in age and the long-term follow-up of 15 years. Our study population was also older than in most other cohorts. The primary outcome was a composite variable, consisting of factors easy to measure and explain to the broad population, and most certainly relevant to patients and caregivers.

## Conclusion

Stroke has a significant impact on cognitive and physical function in stroke survivors above the age of 85. Hence, this reinforces the need for stroke prevention and active long-term rehabilitation to mitigate the consequences of stroke in older people.

## Supplementary Information

**Additional file 1.**

## Data Availability

The data that support the findings of this study are available from ULSAM steering committee ICTUS but restrictions apply to the availability of these data, which were used under license for the current study, and so are not publicly available. Data are however available from the authors upon reasonable request and with permission of ICTUS. ICTUS—Uppsala University, Sweden (uu.se).

## Abbreviations

ANCOVA: Analysis of covariance
BMI: Body mass index
MMSE: Mini-mental state examination
PADL: Personal activities of daily living
PF: Preserved functions
TMT: Trail making test
ULSAM: Uppsala longitudinal study of adult men
