# Supplementary material for: The impact of stroke on the ability to live an independent life at old age: a community-based cohort study of Swedish men
Source: BMC Geriatr. 2023 Mar 6;23:126. doi: 10.1186/s12877-023-03817-1 (PMC9990268; doi:10.1186/s12877-023-03817-1)
Supplement: Supplementary file 1 — Additional file 1. [file 12877_2023_3817_MOESM1_ESM.docx]

**Additional File 1**.*docx.* Supplemental methods with additional data collection information.

**Baseline data collection**

**Education:** Collected through interviews and questionnaire.

Stratified in ordinal categories: <8 years of education

8–12 years of education

>12 years of education

**Living with a partner:** self-administered questionnaire data. Stratified as living alone or with partner.

**Smoking status:** self-administered questionnaire data.

Stratified in ordinal categories: -Current smoking

-Former smoking

-Never smoking

**Leisure time Physical activity:** Self-administered questionnaire data. Patients were asked if:

- They mostly performed sedentary activities (such as crossword-puzzles, TV, or cinema).

- Took regular walks/cycling for pleasure.

- Performed regular physical activity (any sport or heavy gardening) for at least 3 hours/week.

- Performed hard physical training (competitive sports, or high intensity cardio or resistance training).

The answers to the first two questions were stratified as low, the latter two as high physical activity.

**Blood pressure:** A research nurse measured blood pressure in a supine position after 10 mins of rest. The average of two measurements was used.

**High and low density lipoprotein cholesterol**: Fasting blood test. Lipoprotein fractions were acquired with enzymatic techniques using IL Test Cholesterol Trinders's Method and IL Test Enzymatic-colorimetric Method for use in a Monarch apparatus (Instrumentation Laboratories, Lexington, USA). 
High density lipoprotein particles were separated by precipitation with magnesium chloride/phosphotungstate. 
Low density lipoprotein was calculated using Friedewald's formula.

**Body Mass Index (BMI)**: Weight and height measured by a research nurse.

$\frac{weight}{{height}^{2}}=BMI({kg}/{m^{2}})$

**Hypertension**: Defined as a supine blood pressure over 140/90 or prescribed hypertensive treatment, according to the Swedish prescribed drug registry. Anatomic Therapeutic Chemical classification system (ATC) codes: C09A + C09B, C09C +C09D, C08C, C07AB + C07AA, C02CA.

**Diabetes**: Defined as fasting plasma glucose level > 7 mmol/l, glucose above 11.1 mmol/l during glucose intolerance test or prescribed diabetes medication, according to the Swedish prescribed drug registry.

ATC codes: A10A + A10B.

**Atrial fibrillation**: ICD9:427D (427.3). ICD10 (I48) from in-patient registry or atrial fibrillation present in ECG during ULSAM-70.

**Mini-mental state examination (MMSE**)^12^: Cognitive screening test administered by a research nurse.

**Trail making test B(TMT B)**^13^: Pen and paper test administered by a research nurse. Patients are instructed to draw a line linking together alternating numbers and letters. The time until task completion is measured in seconds.

**Enhanced cued recall**^13^**:** A number of pictures on a paper are shown to the test person. After performing a non-related task, the test person is then asked to repeat as many of the pictures as possible.

**Temporal orientation**^13^**:** The test person is asked to state the present date, month, year, weekday, and time of day. Thereafter, a score is assigned depending on the magnitude of errors.

**Charlson comorbidity index**

Index was calculated by an experienced epidemiologist based on the In-patient registry data^14,15^, and the following conditions were scored:

Myocardial infarction

Congestive heart failure

Peripheral vascular disease

Minor stroke or TIA

Dementia

Chronic obstructive pulmonary disease

Connective tissue disease

Peptic ulcer disease

Liver disease: Mild, moderate or severe

Diabetes Mellitus: Uncomplicated or end organ damage

Hemiplegia

Chronic kidney disease

Solid tumor: Localized or metastatic

Leukemia

Lymphoma

AIDS

**Stroke diagnoses**

From the in-patient registry

ICD-8 codes: 430-431 (not 431.91), 433-434.

ICD-9 codes: 430-432 (Not 432.A and 432.B), 434,436

ICD-10 codes: I60-I61 and I63-I64.

**Outcome data collection:**

**Gait speed**^21^**:** Patients were instructed to walk 10 meters at a normal pace. They were timed during the last 6 meters.

**Able to stand without support**^22^**:** Patients tested positive if they managed to stand with feet apart for more than 30 seconds.

**Timed up and go**^23^: Patients are in a seated position. They are instructed to stand up and walk three meters, turn around, walk back and then sit down in the chair again. The whole sequence is timed in seconds.

**MMSE score**^12^: Cognitive screening test administered by a research nurse.

**Enhanced cued recall**^24^**:** A number of pictures on a paper are shown to the test person. After performing a non-related task, the test person is then asked to repeat as many of the pictures as possible.

**Verbal** **fluency score**^24^: Cognitive test administered by a research nurse.

**Geriatric depression Scale-20**^25^: Self-administered symptom scale. A Swedish version of the Geriatric depression scale-15. Five items regarding anxiety, pain, panic, insomnia and hypochondria have been added.

**Quality of life scale**^26^: Participant’s own rating on a vertical 0–100% visual analogue quality of life scale.
